# Supplementary material for: Modeling glioblastoma heterogeneity as a dynamic network of cell states
Source: Mol Syst Biol. 2021 Sep 16;17(9):e10105. doi: 10.15252/msb.202010105 (PMC8444284; doi:10.15252/msb.202010105)
Supplement: Supplementary file 5 — Source Data for Figure 3 [file MSB-17-e10105-s001.zip › Figure3A_sourcedata/GSEA_3065/hallmarks_state6.GseaPreranked.1623416308702/HALLMARK_APOPTOSIS.html]

Details for gene set HALLMARK\_APOPTOSIS[GSEA]

|  || Dataset | state6 |
| Phenotype | NoPhenotypeAvailable |
| Upregulated in class | na\_pos |
| GeneSet | HALLMARK\_APOPTOSIS |
| Enrichment Score (ES) | 0.3021504 |
| Normalized Enrichment Score (NES) | 1.2712729 |
| Nominal p-value | 0.07105263 |
| FDR q-value | 0.23233809 |
| FWER p-Value | 0.829 |
Table: GSEA Results Summary

  

Fig 1: Enrichment plot: HALLMARK\_APOPTOSIS      
 Profile of the Running ES Score & Positions of GeneSet Members on the Rank Ordered List

  

| PROBE | GENE SYMBOL | GENE\_TITLE | RANK IN GENE LIST | RANK METRIC SCORE | RUNNING ES | CORE ENRICHMENT || 1 | HMOX1 |  |  | 0 | 0.545 | 0.0497 | Yes |
| 2 | CDKN1A |  |  | 1 | 0.477 | 0.0932 | Yes |
| 3 | GADD45A |  |  | 2 | 0.419 | 0.1313 | Yes |
| 4 | CCND2 |  |  | 32 | 0.269 | 0.1525 | Yes |
| 5 | FDXR |  |  | 56 | 0.241 | 0.1718 | Yes |
| 6 | BTG2 |  |  | 63 | 0.234 | 0.1925 | Yes |
| 7 | BAX |  |  | 65 | 0.233 | 0.2136 | Yes |
| 8 | PEA15 |  |  | 79 | 0.225 | 0.2326 | Yes |
| 9 | TXNIP |  |  | 176 | 0.183 | 0.2383 | Yes |
| 10 | DDIT3 |  |  | 205 | 0.175 | 0.2510 | Yes |
| 11 | BNIP3L |  |  | 240 | 0.164 | 0.2620 | Yes |
| 12 | GPX4 |  |  | 365 | 0.141 | 0.2606 | Yes |
| 13 | TSPO |  |  | 474 | 0.126 | 0.2596 | Yes |
| 14 | CREBBP |  |  | 484 | 0.125 | 0.2700 | Yes |
| 15 | ATF3 |  |  | 494 | 0.124 | 0.2803 | Yes |
| 16 | SATB1 |  |  | 566 | 0.117 | 0.2827 | Yes |
| 17 | BCL10 |  |  | 588 | 0.115 | 0.2908 | Yes |
| 18 | DAP |  |  | 639 | 0.111 | 0.2952 | Yes |
| 19 | SQSTM1 |  |  | 719 | 0.104 | 0.2956 | Yes |
| 20 | SAT1 |  |  | 744 | 0.102 | 0.3022 | Yes |
| 21 | DAP3 |  |  | 1018 | 0.085 | 0.2785 | No |
| 22 | SOD2 |  |  | 1228 | 0.075 | 0.2612 | No |
| 23 | PMAIP1 |  |  | 1232 | 0.074 | 0.2676 | No |
| 24 | PDCD4 |  |  | 1248 | 0.074 | 0.2726 | No |
| 25 | CCND1 |  |  | 1317 | 0.071 | 0.2712 | No |
| 26 | IFITM3 |  |  | 1398 | 0.068 | 0.2682 | No |
| 27 | CTNNB1 |  |  | 1461 | 0.066 | 0.2670 | No |
| 28 | GADD45B |  |  | 1497 | 0.064 | 0.2688 | No |
| 29 | CYLD |  |  | 1528 | 0.063 | 0.2711 | No |
| 30 | BTG3 |  |  | 2012 | 0.047 | 0.2199 | No |
| 31 | ANKH |  |  | 2098 | 0.045 | 0.2142 | No |
| 32 | BCL2L1 |  |  | 2164 | 0.043 | 0.2106 | No |
| 33 | IRF1 |  |  | 2377 | 0.037 | 0.1895 | No |
| 34 | VDAC2 |  |  | 2516 | 0.033 | 0.1767 | No |
| 35 | PAK1 |  |  | 2589 | 0.031 | 0.1712 | No |
| 36 | DFFA |  |  | 2605 | 0.031 | 0.1723 | No |
| 37 | CASP3 |  |  | 2634 | 0.030 | 0.1718 | No |
| 38 | NEDD9 |  |  | 2666 | 0.030 | 0.1709 | No |
| 39 | RELA |  |  | 2971 | 0.023 | 0.1380 | No |
| 40 | ISG20 |  |  | 3075 | 0.020 | 0.1280 | No |
| 41 | FEZ1 |  |  | 3090 | 0.020 | 0.1283 | No |
| 42 | MCL1 |  |  | 3430 | 0.012 | 0.0904 | No |
| 43 | GSR |  |  | 3436 | 0.012 | 0.0909 | No |
| 44 | CFLAR |  |  | 3461 | 0.012 | 0.0892 | No |
| 45 | CASP6 |  |  | 3477 | 0.012 | 0.0886 | No |
| 46 | HSPB1 |  |  | 3587 | 0.010 | 0.0769 | No |
| 47 | CDC25B |  |  | 3887 | 0.003 | 0.0428 | No |
| 48 | CASP7 |  |  | 3926 | 0.002 | 0.0385 | No |
| 49 | PLPPR4 |  |  | 4336 | -0.006 | -0.0080 | No |
| 50 | RHOT2 |  |  | 4371 | -0.006 | -0.0113 | No |
| 51 | CDKN1B |  |  | 4475 | -0.008 | -0.0224 | No |
| 52 | ROCK1 |  |  | 4476 | -0.008 | -0.0217 | No |
| 53 | ERBB3 |  |  | 4525 | -0.009 | -0.0264 | No |
| 54 | MADD |  |  | 4617 | -0.011 | -0.0358 | No |
| 55 | TIMP3 |  |  | 5017 | -0.019 | -0.0800 | No |
| 56 | SPTAN1 |  |  | 5102 | -0.020 | -0.0878 | No |
| 57 | PPP3R1 |  |  | 5226 | -0.023 | -0.0999 | No |
| 58 | LEF1 |  |  | 5275 | -0.024 | -0.1033 | No |
| 59 | PSEN1 |  |  | 5429 | -0.027 | -0.1184 | No |
| 60 | CASP2 |  |  | 5494 | -0.028 | -0.1232 | No |
| 61 | F2R |  |  | 5881 | -0.036 | -0.1644 | No |
| 62 | ENO2 |  |  | 5889 | -0.036 | -0.1619 | No |
| 63 | DPYD |  |  | 5951 | -0.037 | -0.1656 | No |
| 64 | SC5D |  |  | 6168 | -0.042 | -0.1866 | No |
| 65 | PSEN2 |  |  | 6403 | -0.047 | -0.2092 | No |
| 66 | ETF1 |  |  | 6699 | -0.056 | -0.2381 | No |
| 67 | DNAJA1 |  |  | 6816 | -0.059 | -0.2461 | No |
| 68 | PTK2 |  |  | 6894 | -0.062 | -0.2493 | No |
| 69 | ADD1 |  |  | 6959 | -0.063 | -0.2509 | No |
| 70 | MMP2 |  |  | 7016 | -0.065 | -0.2513 | No |
| 71 | PPT1 |  |  | 7033 | -0.066 | -0.2472 | No |
| 72 | TGFB2 |  |  | 7048 | -0.066 | -0.2428 | No |
| 73 | SOD1 |  |  | 7066 | -0.067 | -0.2386 | No |
| 74 | BMP2 |  |  | 7299 | -0.074 | -0.2585 | No |
| 75 | RHOB |  |  | 7306 | -0.075 | -0.2524 | No |
| 76 | SLC20A1 |  |  | 7392 | -0.078 | -0.2551 | No |
| 77 | XIAP |  |  | 7394 | -0.078 | -0.2482 | No |
| 78 | ERBB2 |  |  | 7405 | -0.078 | -0.2422 | No |
| 79 | TOP2A |  |  | 7430 | -0.079 | -0.2378 | No |
| 80 | JUN |  |  | 7513 | -0.082 | -0.2398 | No |
| 81 | CASP4 |  |  | 7545 | -0.084 | -0.2357 | No |
| 82 | EBP |  |  | 7564 | -0.084 | -0.2301 | No |
| 83 | LGALS3 |  |  | 7566 | -0.084 | -0.2225 | No |
| 84 | BID |  |  | 7709 | -0.092 | -0.2305 | No |
| 85 | TNFRSF12A |  |  | 7784 | -0.094 | -0.2304 | No |
| 86 | TIMP2 |  |  | 7809 | -0.096 | -0.2244 | No |
| 87 | IFNGR1 |  |  | 7892 | -0.101 | -0.2247 | No |
| 88 | CDK2 |  |  | 8018 | -0.108 | -0.2292 | No |
| 89 | WEE1 |  |  | 8054 | -0.111 | -0.2231 | No |
| 90 | CAV1 |  |  | 8156 | -0.117 | -0.2240 | No |
| 91 | IGF2R |  |  | 8211 | -0.122 | -0.2191 | No |
| 92 | LMNA |  |  | 8225 | -0.124 | -0.2093 | No |
| 93 | GSN |  |  | 8339 | -0.137 | -0.2098 | No |
| 94 | DNM1L |  |  | 8350 | -0.139 | -0.1983 | No |
| 95 | CLU |  |  | 8387 | -0.145 | -0.1893 | No |
| 96 | HMGB2 |  |  | 8388 | -0.145 | -0.1761 | No |
| 97 | TAP1 |  |  | 8393 | -0.146 | -0.1633 | No |
| 98 | DNAJC3 |  |  | 8407 | -0.147 | -0.1513 | No |
| 99 | EMP1 |  |  | 8641 | -0.209 | -0.1591 | No |
| 100 | ANXA1 |  |  | 8651 | -0.213 | -0.1407 | No |
| 101 | BRCA1 |  |  | 8659 | -0.217 | -0.1217 | No |
| 102 | BCAP31 |  |  | 8710 | -0.248 | -0.1049 | No |
| 103 | TIMP1 |  |  | 8751 | -0.290 | -0.0831 | No |
| 104 | APP |  |  | 8758 | -0.300 | -0.0564 | No |
| 105 | PLAT |  |  | 8772 | -0.327 | -0.0281 | No |
| 106 | CD44 |  |  | 8778 | -0.337 | 0.0021 | No |
Table: GSEA details [plain text format]

  

Fig 2: HALLMARK\_APOPTOSIS: Random ES distribution      
 Gene set null distribution of ES for **HALLMARK\_APOPTOSIS**
